# A Bandwidth-Efficient Service for Local Information Dissemination in Sparse to Dense Roadways

**DOI:** 10.3390/s130708612

**Published:** 2013-07-05

**Authors:** Estrella Garcia-Lozano, Celeste Campo, Carlos Garcia-Rubio, Alberto Cortes-Martin, Alicia Rodriguez-Carrion, Patricia Noriega-Vivas

**Affiliations:** Department of Telematic Engineering, University Carlos III of Madrid, Avda. de la Universidad 30, Leganes 28911, Madrid, Spain; E-Mails: celeste@it.uc3m.es (C.C.); cgr@it.uc3m.es (C.G.-R.); alcortes@it.uc3m.es (A.C.-M.); arcarrio@it.uc3m.es (A.R.-C.); pnoriega@it.uc3m.es (P.N.-V.)

**Keywords:** VANET, service discovery, bandwidth efficiency, multi-hop broadcast, store-carry-forward

## Abstract

Thanks to the research on Vehicular Ad Hoc Networks (VANETs), we will be able to deploy applications on roadways that will contribute to energy efficiency through a better planning of long trips. With this goal in mind, we have designed a gas/charging station advertising system, which takes advantage of the broadcast nature of the network. We have found that reducing the number of total sent packets is important, as it allows for a better use of the available bandwidth. We have designed improvements for a distance-based flooding scheme, so that it can support the advertising application with good results in sparse to dense roadway scenarios.

## 1. Introduction

Thanks to the current interest in intelligent transportation systems (ITS) and the efforts from several governments and the research community, roadways are to become connected networks. Vehicles will be able to communicate with each other and with roadside units. This capability will allow the deployment of applications that contribute to energy efficiency and the reduction of CO_2_ emissions. These goals can be achieved through a better planning of stops on a long trip.

We have designed a “push” service discovery system that lets gas stations advertise their location in a wide enough radius. This way, we make this information available to all the vehicles in the area, and they can use it for planning the best next stop. We take advantage of the wireless, broadcast nature of IEEE 802.11p communications. A few selected vehicles forward the message, so that all those in the target area receive the information.

Using an efficient flooding scheme for this task is a key feature. By efficiency, we refer to the minimum use of the shared bandwidth, as it is a limited good in the VANET. We have optimized the formulation of a forwarding scheme we had already proposed in [1], in order to reach this goal. In addition, we have created a store-carry-forward mechanism adapted to this scheme, so that the service works in not fully connected roadways. By fully connected, we mean that a new relay exists at every hop, so that there is a path from the origin to the destination point.

The result is a gas/charging station advertising solution for roadway environments. It would run as a UDP application in the vehicle's on-board computer and in roadside units owned by gas stations. We assume that both are equipped with IEEE 802.11p and GPS capabilities. The on-board computer will select from the incoming advertisements only the ones that fit the planned route best and present a sorted list to the driver according to her preferences (price, affiliation, *etc.*). The algorithm for selecting and sorting the notifications is out of the scope of this article, so we leave it for future work.

The article is organized as follows: First, in Section 2, we present a context for our contribution from the perspectives of service discovery and multi-hop broadcast in VANETs. Next, we explain the basis of the new service and how it works in Section 3. In Section 4, we describe the flooding optimization. In Section 5, we introduce the new store-carry-forward mechanism. Next, in Section 6, we explain the final message format. In Section 7, we test, by simulations, the service described in the leading ones. We end the paper with the conclusion and ideas for future work in Section 8.

## 2. Related Work

Location-aware services have been receiving a lot of attention in the last few years, and the research community is working on different ways to provide them. For example, the work in [2] proposes a proactive contextualization by means of prediction. A lightweight application that resides in the user's phone constantly learns her mobility pattern according to the GSM cells to which it registers. By means of LZ-based algorithms, it is able to predict future locations and, hence, be aware of the future context. In the same article, the authors present an application for recommending bus lines to the user based on their findings. In contrast to this approach, we are providing a context in a reactive way, once the user is in the service influence area.

An application more related to our solution is the gas station recommender proposed in [3]. Vehicles obtain information about gas stations and compute recommendations based on the context (fuel level, location, *etc.*). One of the options they suggest is that, when a driver pays at a gas station, the vehicle learns (automatically or not) the prices and other data and disseminates them into the VANET. It is not clear how the information is forwarded, either.

The usual way to implement service discovery in a bandwidth-efficient way is to rely on roadside routers, being in the form of service directories or mere backbones. The work presented in [4] is a good example of this. Depending on infrastructure may give some advantages, like bandwidth usage optimization. However, on the other hand, it also takes autonomy away from the system. There might not be roadside units other than the ones installed by the gas station owners. In addition, having a service directory is not necessary for a “push” discovery, as we intend to do. A well-known service discovery platform that happens to support the “push” mode is the Vehicular Information Transfer Protocol (VITP) [5]. This proposal assumes that vehicles perform geographic routing and then flood the information into the target area. The authors also consider that flooding all the way to the target area could be of interest. We intend to improve the bandwidth usage by means of a distance-based broadcast.

Non-safety applications, like the one we present here, do not require a very short reaction time. This means we can adapt our strategy, so that we can reduce the number of packet retransmissions. We can find the same motivation and a similar approach to our solution in [6]. There is a variety of proposals in the literature for achieving an efficient multi-hop broadcast in a vehicular environment. For example, the work in [7] agrees with the results we reached in [1]. In this article, the authors evaluate several flooding schemes and conclude that the distance-based solution offers the best performance. They propose a time-slotted solution, named Slotted 1-Persistence Broadcasting. We, on the other hand, are going to propose here a continuous time approach in order to minimize the probability of collisions. Another distance-based solution can be found in [8]. The authors present the Geographical Data Dissemination for Alert Information (GEDDAI). They add the notion of “sweet spots”, which are zones in the coverage radius of a transmitting vehicle. Vehicles located in such sweet spots get preference in forwarding over the others, because they are supposed to reach a minimum overlapping section of the road. We find that this idea is useful only for very wide highways, where the transmission range does not cover the road width. In most of the current interurban roads, our proposal would be more than enough. The value of the coming angle for the optimization of the bandwidth usage is also used in [9].

We are going to use the store-carry-forward paradigm to tackle the occasional disconnections in sparse roadways. According to it, moving relays in a sparse network are forced to store the message until they find a new neighbor to pass it to. The actual implementation of store-carry-forward is highly dependent on the general forwarding protocol design. This paradigm has proven its effectiveness and is widely used in the VANET scenario; some examples are [10–12].

In [10,11], all the vehicles exchange frequent beacons with information about their location. They use the resulting database to decide if they should forward a new message, store it or simply drop it. In [10], a vehicle that receives a new message checks whether it knows of any neighbor in its own lane in the direction of the message. If there are not any, it checks if there is someone in the opposite lane. If there are none either, it stores the message and waits until it receives a beacon from someone in one of those two situations. The same approach is used in [11]. Every vehicle has a table of one-hop neighbors with their location information. When it hears a new message, it crosses out from that list every neighbor that must have heard the message, too. It does the same with eventual duplicates. If after this procedure there are not any neighbors left in the list, it stores the message. The vehicle will forward it as soon as there is a new neighbor in the list that has not been able to hear the message.

In [12], vehicles do not exchange beacons. In this solution, the message is forwarded via unicast, and each new relay is selected by the previous one. When a vehicle receives a message that should be forwarded, it asks all the possible neighbors that are in its coverage area about their location. With their answers, it computes the one that is closer to the destination in the predefined path. If the neighbors are in fact in a worse locationthan its own, it keeps the message and tries again later (in the paper it is not specified when). As we will explain later, our implementation is closer to this one in the sense that we do not make use of a neighbor database, either. However, our approach, on the other hand, is totally different in how the selection of the relay and the decision of storing the message are done.

## 3. The Advertising System

First, it is important to highlight the difference between city and roadway scenarios when choosing a strategy for a given task. What is best for one may have a poor performance in the other. In a roadway, the path is almost linear, and there are few intersections. For now, we have focused on this type of scenario. We consider roadways in which there is a specific traffic density, *ρ*, in each direction at a given time. A direction's density may be zero if the roadway is one-way only or if there are not any vehicles in that direction at the moment. We also expect a maximum legal speed, *v_limit_*.

Next, there are two types of service discovery. In the “push” mode, the service providers send their information to everyone. In the “pull” mode, it is the node interested in a service that looks for a provider. In a broadcast environment, such as a VANET, it makes sense to use the “push” mode to advertise gas stations (or any other generally interesting roadside service).

We assume that vehicles have GPS and 802.11p communication capabilities. Furthermore, they have an on-board computer that can display a list of the gas stations that fit the planned route best, sorted according to the driver's preferences (as price or affiliation). Gas stations can send messages by means of a roadside unit (RSU) of their own. RSU is the common name for fixed infrastructure in a vehicular networking scenario.

**Figure 1.**
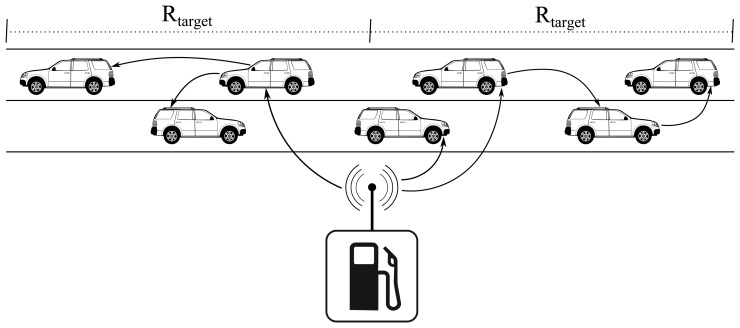
Scenario representation.

The vehicles, as well as the gas station's RSU are able to run our solution, designed as an application over UDP. Their antennae have a coverage range of radius, *r*, which is expected to be of just a few hundred meters (200–500 m). Therefore, when the gas station sends a message, it reaches only those vehicles that are closest to it. They are responsible for forwarding the message to the next group in each direction. Those must forward the message again, and so on, until it has reached the edge of the target area and about every vehicle in it has heard it. A visual representation is in Figure 1.

Gas stations advertise their location and other data periodically. The spread message contains the gas station location and a target zone. The location could be manually configured by the manager if it cannot be obtained automatically by a GPS device. The target zone is determined by a radius of interest, *R_target_*. This and the time interval between announcements are both configurable. The manager of the gas station is supposed to have a PC or mobile application that lets her adjust the settings and transfer them to the RSU.

In addition to such basic information, the gas station can also insert other data in the message, such as the brand name, prices, special deals, *etc*. There are also other fields related to the dissemination procedure, which will be explained later. The final message format is described in more detail in Section 6.

In the following sections, we are going to explain specific aspects of how the service works and test them in a sample road scenario through simulations.

## 4. Distance-Based Forwarding for Efficiency

As stated before, a key necessity of our advertising system, which works in “push” mode, is to be bandwidth-efficient. At the same time, we want these messages to reach all the vehicles in the target zone, so we need to perform a broadcast.

In a former work [1], we tested the performance of several basic broadcasting schemes. Using prior simulations as a starting point, we concluded that distance-based flooding provides the best performance in terms of bandwidth usage and proposed a specific algorithm. Here, we optimize the formulation and propose specific values for its configuration. In addition, we provide a formal analysis of the ratio of forwarders per receiver that makes this scheme the best option for all-purpose broadcasting.

### 4.1. Description

In distance-based flooding, the most distant node from the last relay gets the highest priority. We give the priority by means of an order for forwarding.

When a node receives a new packet, it launches a short wait, *W*, in which it expects to receive a potential duplicate from another relay. If it hears any, it saves the distance to the nearest node from which it received the same packet. Next, it calculates a forwarding delay, *t_w_*, as:

(1) $$t_{w}=T_{\text{max}}\times (1-\text{min}\{\left|d(x,x_{\text{relay}})\right|\}/r)$$

where *T_max_* is the (configurable) maximum wait and *r* is the coverage radius.

When the second timer (*t_w_*) expires, it forwards the message. This way, the most distant node has the shortest wait. Unlike other proposed distance-based algorithms, such as Slotted 1-persistence in [7], we use a continuous time expression, in order to minimize collisions. When the most distant node forwards the packet, the rest hear it and abort their forwarding procedure.

### 4.2. Ratio of Forwarders per Receiver

Ideally, this scheme chooses only one forwarder from every new covered area. However, it may happen that two nodes are located so close that they calculate similar forwarding delays. When their timers expire, they both try to retransmit the packet almost simultaneously. As the second node is not able to hear the first one's packet before forwarding, this results in a collision.

We call *τ* the minimum difference between the delays calculated by two consecutive nodes, so that this situation does not occur. We explain the concept of *τ* in more detail in Section 7.1.1.

Let us assume that the vehicle density is enough, so that there is not any disconnection in the distance, *R_target_*, of interest. We say that the number of receivers in such a distance is *ρ* × *R_target_*, where *ρ* is the traffic density in the road segment in vehicles per space unit.

If there is not any collision, only one node from every covered area forwards the message. Typically, this node is not at the end of the radius, but at a distance, *E*[*d_max_*]. This is the average distance at which the most distant node is from the last relay within the coverage area.

For simplicity, if we model the highway as an unidimensional, straight scenario, we can say that the position of each vehicle in it is an event from a Poisson process. Hence, we can model the distance between nodes as following an exponential distribution with *λ* = *ρ*. Given that this distribution is memoryless, *E*[*d_max_*] will be the maximum possible distance between sender and receiver, *r*, minus the average distance between cars, 1/*ρ*: *E*[*d_max_*] = *r* − 1/*ρ*.

The total number of nodes that forward the message in the given distance, *R_target_*, will be as many as the covered zones. Therefore, the ideal ratio would be:

(2) $$\frac{\frac{R_{\text{target}}}{E[d_{\text{max}}]}}{\rho \times R_{\text{target}}}=\frac{\frac{R_{\text{target}}}{r-1/\rho }}{\rho \times R_{\text{target}}}=\frac{1}{\rho r-1}$$

However, as we have already said, there can be more than one sender per covered area. This is the case when two consecutive nodes have a difference between their respective forwarding delays (*t*_1_ and *t*_2_) less than *τ*:

(3) $$t_{2}-t_{1}<\tau \to T_{\text{max}}\left(1-\frac{d_{2}}{r}\right)-T_{\text{max}}\left(1-\frac{d_{1}}{r}\right)<\tau \to d_{1}-d_{2}<\frac{r\tau }{T_{\text{max}}}$$

Therefore, now, we know the average number of nodes that actually forward the message per covered zone and the true ratio of forwarders per receiver:

(4) $$\left(1+(r\tau /T_{\text{max}})\times \rho )/(\rho r-1\right)$$

While *ρrτ*/*T_max_* < 1, the maximum ratio will be less than 2/(*ρr* − 1), that is, two nodes per covered area. Given that the forwarders are always the closest ones to the edge, the number of coverage areas in the distance, *R_target_*, is minimized. The result is, as we had previously found by testing in [1], that this scheme needs the minimum number of retransmissions to reach 100% of the cars.

### 4.3. Choosing the Maximum Per-Hop Delay

From Equations (1) and (4), the reader can notice that *T_max_* plays an important role in the performance of the scheme. The lower this value, the shorter the per-hop delay in Equation (1) will be. However, we want to keep it high enough, so that the ratio in Equation (4) is as low as possible.

We consider that the application should be able to configure *T_max_*. However, we can give some hints for choosing a good value.

First, according to the reasoning in the previous section, the minimum value should be, for the highest *p* expected in the road, *T_max_* > *ρrτ*.

Now, we want to recommend a value for this parameter. As the forwarding ratio also depends on the traffic density, we can only express it as a function of *ρ*. The traffic density is time-variant and space-variant. We could determine the density at a high-level for a road segment, but a given vehicle may be experiencing a different density locally. Even more, this same vehicle may experience a very different traffic situation after a few seconds. Therefore, our aim is to suggest a value for *T_max_* that would yield good results for any value of *ρ*.

In Equation (4), *τ*/*T_max_* is multiplied by *ρr* and divided by *ρr* − 1. Let us fix the value of *ρ. τ* and *τ* are already constants. If *T_max_* is the only variable of the equation, different values of *ρ* will provide very similar curves at different heights. In case the nodes cannot learn the actual road density, they can use a default value and still expect good results.

For any given *ρ*, we select the point from which we do not experience a significant improvement in the forwarding ratio. As subjective as this statement is, the application could also choose where to put the limit. We will use a specific one for the reasoning that follows.

If we fix *ρ* in Equation (4), the expression is a function of *T_max_* in the form, *y* = 1/*x*. The curve, 1/*x*, generates a cone around the axial symmetry axis in *y* = *x* with the vertix in (1, 1). Before this point, the function goes down very fast and, from then on, very slowly, being lim*_x_*_→∞_ 1/*x* = 0.

The zone of interest in our problem is at the right of the symmetry axis. What is more, we can take this intersection as a reference. From *x* = 1 until *x* → ∞, the curve goes down from *y* = 1 until *y* = 0. We search, within this zone, the *x* where the curve reduces its *y* value by 95%, that is, 1/*x*_95%_ = 0.05 → *x*_95%_ = 1/0.05.

Now, to translate it to our equation, we only take into account the transformations that imply a change in the proportion of the curve:

(5) $$T_{\text{max}}=\frac{\rho r\tau }{\rho r-1}\times x_{95\%}$$

As we want a good value for any given *ρ*, we can simply find the limit: lim*_ρ_*_→∞_ *T_max_* = *τx*_95%_.

## 5. How to Solve Cases of Low Traffic Density

When the roadway is sparse (*i.e.*, during non-rush hours) we find gaps between vehicles usually larger than the coverage radius. We need a mechanism to overcome this situation and let the message travel through the whole area of interest. Therefore, we have added our own store-carry-forward mechanism, customized to the previously described scheme. The basic idea in store-carry-forward is that, if a relay cannot find another one to forward the message, it stores the message and carries it for some time, until it finds someone who can forward it. Now, we describe a series of measures to implement this idea successfully in our forwarding scheme for the application scenario.

### 5.1. Finding the Right Time to Forward Again

Given the case that a vehicle has to store-carry-forward a message, we decided to avoid using *hello* packets to detect new neighbors. The emission of periodic packets from every vehicle would increase the channel load highly, and one of our goals is to minimize the channel usage this service needs. Therefore, if a vehicle cannot detect a new vehicle when it enters its coverage range, it can only forward it periodically in the hope that it will meet someone new at some time.

If the frequency of retransmissions is too high, there will be little variation in the scenario, and the probability of finding new neighbors will be low. If the time period is too long, the relay may be missing passing vehicles while it is waiting. Therefore, we have to maximize the probability of finding new neighbors at the time of forwarding.

The chosen time period for this task will be an estimate of how long it will take this vehicle to get out of its currently covered area. That is:

(6) $$r/v_{\text{limit}}$$

We use the legal speed limit, *v_limit_*, to avoid assumptions about the average speed on this road. If the vehicle is traveling at a lower speed than the limit, it will forward the message again before it has left the last covered area. However, the rest of the vehicles in its own direction, or the vehicles in the opposite direction, may be traveling at a speed closer to the limit, and so, they will not miss the retransmission.

**Figure 2.**
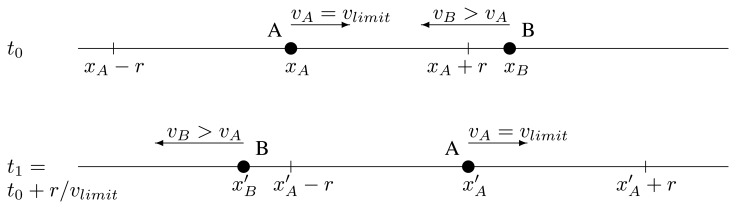
Case depiction.

Let us consider the situation represented in Figure 2. The relay, denoted by point *A*, forwards a message at *t*_0_. The vehicle coming in the opposite direction (*B*) does not hear it, because it is further than *r* from *A. A* travels at the speed limit, while *B* travels at a higher speed. At *t*_1_, when *A* forwards once more, *B* will be again further than *r*, but past the relay this time. This case is possible, but not very probable. Furthermore, the reception of the message is not vital. The information in it is not safety-related, and there are still traffic signs on the roadway. Therefore, we opt to not rise the frequency of retransmissions, at the price of this possibility.

### 5.2. Selecting a Relay

We must provide some mechanism, so that the relay can learn if the forwarding was successful or if it has to carry the message and forward it again in the future. The easiest way, which avoids putting more packets into the channel, is awaiting a new retransmission from the next relay If this does not occur, it means that nobody has become the next relay, and hence, the last one must activate the store-carry-forward scheme.

However, this implies a problem: if the new relay gets out of the coverage area of the last one before the retransmission, the latter will not be able to detect it. Then, it will go on forwarding over and over again. As the rest of the vehicles have already heard the message, they will not become a new relay of an old packet. Therefore, it will only stop when it reaches the border of the message target radius.

To avoid this situation, we add a restriction over the group of vehicles in the coverage range of a relay. Any vehicle will be eligible to be the next relay if it is at *x* < *r* − *d_guard_*. This means that we set a guard distance (*d_guard_*) to prevent vehicles close to the edge of the coverage range from taking part in the forwarding process. This distance, *d_guard_*, is given by the vehicles' direction and speed and a set of times: the maximum time it takes the network to process the packet (collision resolution and propagation); the initial wait, *W*, of the forwarding scheme; and the maximum wait, *T_max_*, given by the distance function. They all account for Δ *t_max_*.

**Figure 3.**
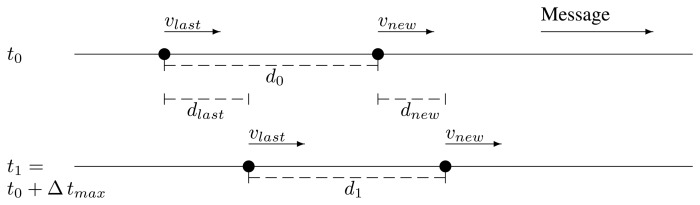
Case 1 depiction.

**Figure 4.**
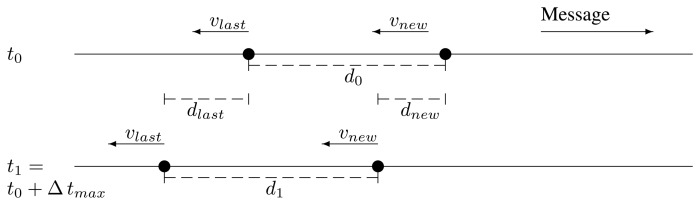
Case 2 depiction.

**Figure 5.**
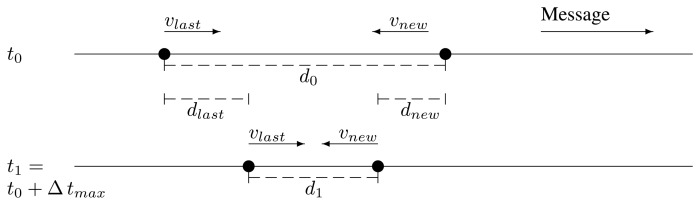
Case 3 depiction.

**Figure 6.**
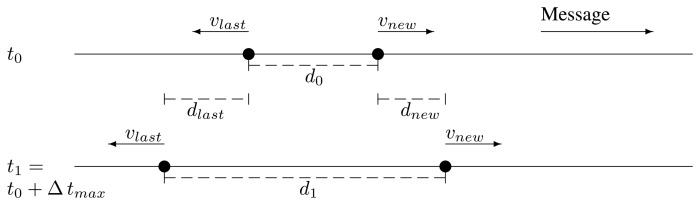
Case 4 depiction.

There are four possible situations:

1. The last relay and the potential new one are traveling in the same direction and in the same direction as the message, too. Their respective current speeds are *v_last_* and *v_new_*. In the moment of forwarding, *t*_0_, they are apart a distance, *d*_0_. After the maximum time it may take a retransmission by the new one to reach the last one, Δ *t_max_*, the last relay has traveled a distance, *d_last_*, and the potential new relay, a distance *d_new_*. They are apart a distance, *d*_1_, depicted in Figure 3:
  $$d_{1}=d_{0}-d_{\text{last}}+d_{\text{new}}=d_{0}+\Delta t_{\text{max}}(v_{\text{new}}-v_{\text{last}})$$
  This *d*_1_ must be less than the coverage radius, *r*. Therefore, if *v_new_* > *v_last_*, we may face a case where *d*_1_ becomes greater than *r*.
2. The last relay and the potential new one are traveling in the same direction, but in the opposite direction as the message. We can see an illustration of this in Figure 4. In this case, the distance, *d*_1_, after Δ *t_max_* is:
  $$d_{1}=d_{0}+d_{\text{last}}-d_{\text{new}}=d_{0}+\Delta t_{\text{max}}(v_{\text{last}}-v_{\text{new}})$$
  Now, the problem may appear when *v_new_* < *v_last_*.
3. The last relay and the potential new one are traveling in opposite directions, coming closer. Then, it is impossible that they get out of range of the other. This case is shown in Figure 5.
4. The last relay and the potential new one are traveling in opposite directions, getting further. This case is depicted in Figure 6. The maximum distance after the new retransmission is:
  $$d_{1}=d_{0}+d_{\text{last}}+d_{\text{new}}=d_{0}+\Delta t_{\text{max}}(v_{\text{last}}+v_{\text{new}})$$
  Furthermore, no matter which speed is higher, *d*_1_ may become greater than *r*.

Because of the forwarding scheme, the location of the last relay must be present in the message. In order to make the new relay able to avoid a problematic case, we are going to include the current speed, too. However, to preserve some of the driver's privacy, we do not want to include whether the vehicle is going towards the RSU or away from it. Therefore, the new potential relay will use the most restrictive rule to decide if it is eligible as a relay or not:

(7) $$x<r-\Delta t_{\text{max}}(v_{\text{last}}+v_{\text{new}})$$

If this inequation is true, the vehicle that heard the message will initiate the scheme described in Section 4 to compete for becoming the new relay. For speeds of 120 km/h, that would mean a reduction of less than 6 m on the coverage radius. This limitation lowers the effective vehicle density for forwarding. However, it avoids the wrong activation or the wrong lack of deactivation of the store-carry-forward mechanism, which would cause a high number of unnecessary retransmissions.

### 5.3. Resolution in the Case that More than One Relay is Elected

After forwarding, the relay waits for a new retransmission from someone else before repeating the message itself. If it hears another vehicle doing so, it deactivates the store-carry-forward mechanism. However, we know from Section 4.2 that more than one vehicle may try to forward at almost the same time. After the network processes the collision, all the duplicates reach all the vehicles that forwarded the message simultaneously, because they are a short distance from each other. As every forwarder hears a duplicate of the message after the retransmission, they may all believe that there is a new relay. Maybe, in fact, their duplicates did not reach any other new vehicle, so the dissemination would end there.

We avoid this effect by adding a Time To Live (TTL) field in the message. When a forwarder receives a message with the same TTL, it knows it is a duplicate from a vehicle nearby. It does not mean that there is a new relay.

Now, who must activate store-carry-forward if it is necessary? The answer is, the vehicle that is furthest from the RSU, whose location is in the message. Upon every duplicate, every relay can find out if they are more apart from the RSU than the sender.

### 5.4. Carrying the Message Away from the RSU

While the relay is carrying the message, it keeps traveling in its own direction. If it goes in the opposite direction as the message, the latter would travel backwards without any guarantee of getting further thanks to the next relay. Hence, it is not advisable that any vehicle, regardless of its direction, could carry the message.

As we do not make use of beacons, a vehicle cannot know beforehand when it will find a sparse network situation. Even in an area with a medium vehicle density, we can find a sparser zone just a few hundred meters ahead; for example, after a way out to an important roadway. It would make sense that only those traveling in the same direction as the message can be the next relay. However, we cannot depend on just the vehicles that travel away from the gas station. They could be very few or even nonexistent if the roadway has only one direction.

What we do, then, is allow vehicles that travel towards the gas station to be relays. However, if they have to store, carry and forward the message, vehicles traveling in the opposite direction must help, even if the message is old to them.

Note that all vehicles maintain a duplicate table, so that they do not forward old messages. If a vehicle traveling away from the gas station hears a message that a vehicle in the other direction is carrying, it is bound to be old to it, because this vehicle comes from the message's point of origin.

To solve this problem, we set a flag in the message to indicate whether the relay is doing store-carry-forward “backwards”, *i.e.*, in the opposite direction as the message should travel.

If the flag is set to one, a vehicle that travels away from the gas station's RSU will act as if the message is new to it, even if it is not. It may become the new relay, “rescue” the message and carry it in the right direction again.

### 5.5. Selecting the Best “Rescuer” in Backwards Store-Carry-Forward

**Figure 7.**
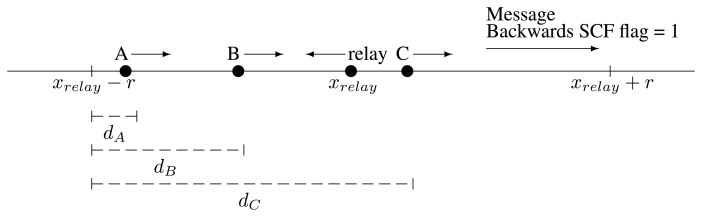
Selection of the best “rescuer”.

When a vehicle hears a message with the backwards flag set to one, it knows it is a special situation. The message cannot travel in the intended direction. In such a case, any vehicle traveling in the same direction as the message must help. It will act as if the message was new, even if it is not. In such a case, this vehicle and other potential “rescuers” can be in any point of the range, ±*r*, from the relay location, *x_relay_*, as depicted in Figure 7. Therefore, instead of using Equation (1), the “rescuers” will use a variation.

First, they will compute the distance to *x_relay_* − *r* instead of the distance to *x_relay_*. Then, they will consider a maximum distance of 2*r* between the closest and the furthest from the origin. The maximum contention time, *T_max_*, must always be the same. All this is reflected in Equation (8):

(8) $$t_{w}^{\prime }=T_{\text{max}}\times (1-d(x,x_{\text{relay}}-r)/2r)$$

Therefore, this new $t_{w}^{\prime }$ will be the time computed for the contention by the potential “rescuers” in the case of backwards store-carry-forward.

### 5.6. Detection of the New Relay by All the Neighbors

**Figure 8.**
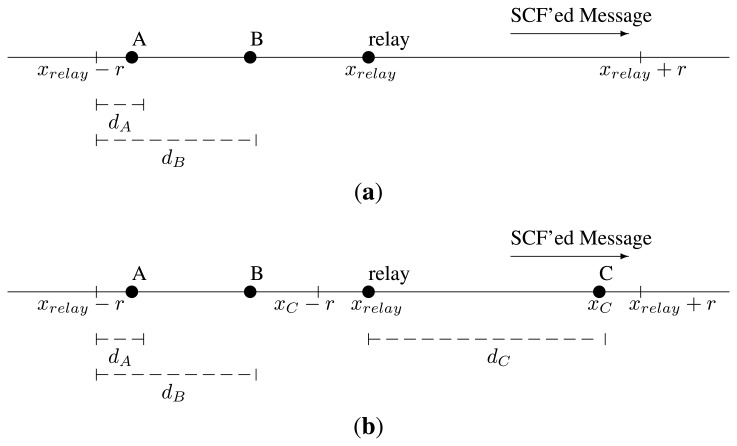
Relay detection in the range [*x_relay_* − *r*, *x_relay_* + *r*]. (**a**) Equation (9) lets us select B as a new relay; (**b**) B and C are both selected as new relays. The previous relay must repeat the message from C, so that B can hear it.

When a vehicle is doing store-carry-forward, may it be backwards or onwards, it will eventually find a new set of neighbors. They can be located at any point in the range, ±*r*, from its location (*x_relay_*). Let us depict the case with Figure 8(a). Because there is no one in the segment, (*x_relay_*, *x_relay_* + *r*], the new relay must be chosen from those in the segment, [*x_relay_* − *r*, *x_relay_*). This would be the one that is closest to the relay, instead of the furthest. That is, if a vehicle hears a new message from a vehicle that is further from the origin than itself, it will still try to forward it, but using this other form of Equation (1):

(9) $$t_{w}^{\prime \prime }=T_{\text{max}}-(1-d(x,x_{\text{relay}}-r)/r)$$

The one who will forward and become the new relay is the vehicle most apart in the direction of the message, (*x_relay_*, *x_relay_* + *r*]. If there are not any vehicles in that range or if the vehicles in the range [*x_relay_* – *r*, *x_relay_*) are not able to hear it, they will select a new forwarder among themselves. This situation is depicted in Figure 8(a).

The existence of an unnoticed vehicle in the range, (*x_relay_*, *x_relay_* + *r*], poses a problem. This case is shown in Figure 8(b). Another vehicle from the range, [*x_relay_* − *r*, *x_relay_*), may be further than *r* from the new relay. Because it did not hear it, it will forward the message. In addition, it will have to store-carry-forward, because it will not find any other one to become the relay of an old message.

In order to solve this conflict, the last relay will repeat the message as soon as it hears it if the new relay is in (*x_relay_*, *x_relay_* + *r*].

As explained in Section 5.3, when a forwarder hears another one retransmitting the message with the same TTL, it compares its distance to the origin with the other's. Now, the forwarder from the range, [*x_relay_* − *r*, *x_relay_*), will hear the retransmission from the previous relay, located in *x_relay_*. Therefore, it will not try to store-carry-forward it.

The new relay in (*x_relay_*, *x_relay_* + *r*] will hear, at least, the repetition of its own message by the previous relay. Any duplicate coming from a point closer to the origin must be ignored, then. However, this new relay may be traveling towards the origin and be closer to it when it hears the repeated message. It cannot tell if the message is a repetition of its own one or not, because the packet does not contain any vehicle identifier. Therefore, the new relay will use a margin of *v* × *W* to ignore such a case, where *v* is its current speed.

### 5.7. Stopping Conditions

Lastly, we need a stop condition for two different situations. One of them occurs when, despite the preventive measures, the next relay travels very fast and the previous one cannot hear the new retransmission. Then, the latter will find itself forwarding forever, because, as noted above, the message is old for the rest of the vehicles. The other situation happens in a roadway with a very low vehicle density. The vehicle cannot find any other one traveling in the same direction for a long time. This would be the case, for example, at 2 am in a weekday. Though increasing the channel load would not be a problem in this case, the message becomes meaningless after the vehicle leaves the target radius.

We will force the vehicle to stop doing store-carry-forward if it gets out of *x_origin_* ± *R_target_*.

### 5.8. Resulting Algorithm

To summarize the algorithm, we offer Procedures *receive_message*() and *timer_expired*(). Here, we take the RSU location as the coordinates origin. The value of *x* is the distance to the RSU, regardless of the sense or the real orientation.


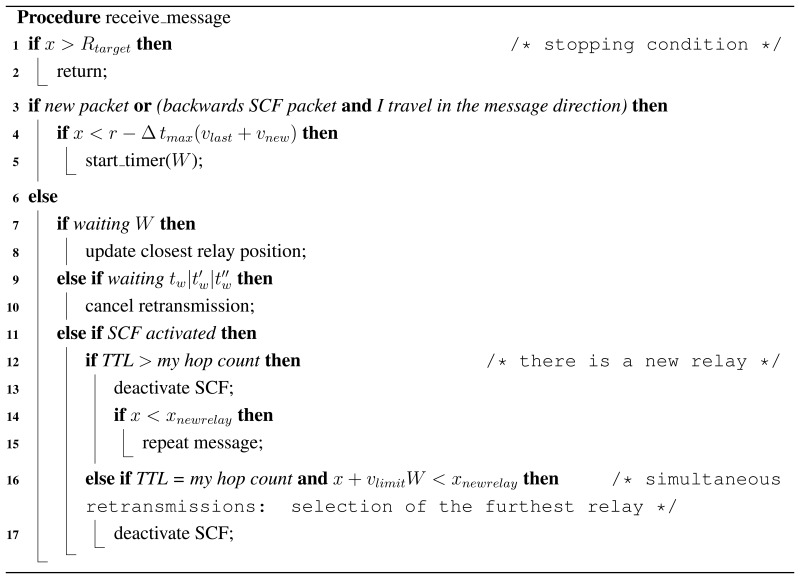


## 6. Message Format

Along with the specific information (brand name, prices, *etc.*) that the service provider wants to spread, the station location and the target zone must be specified in the message. The provider location is unique and fixed, so it is used to detecting duplicates. The (configurable) target zone is determined by simply indicating a radius of interest. It is also necessary to replace the location and the speed of the last forwarder at every hop, so that we can make use of our distance-based flooding scheme and the store-carry-forward mechanism. A “backwards SCF” flag is also necessary, so that vehicles traveling in the opposite direction as the message can signal when they are doing store-carry-forward. Finally, a TTL field is used to determine the occurrence of simultaneous duplicates by all the involved vehicles.


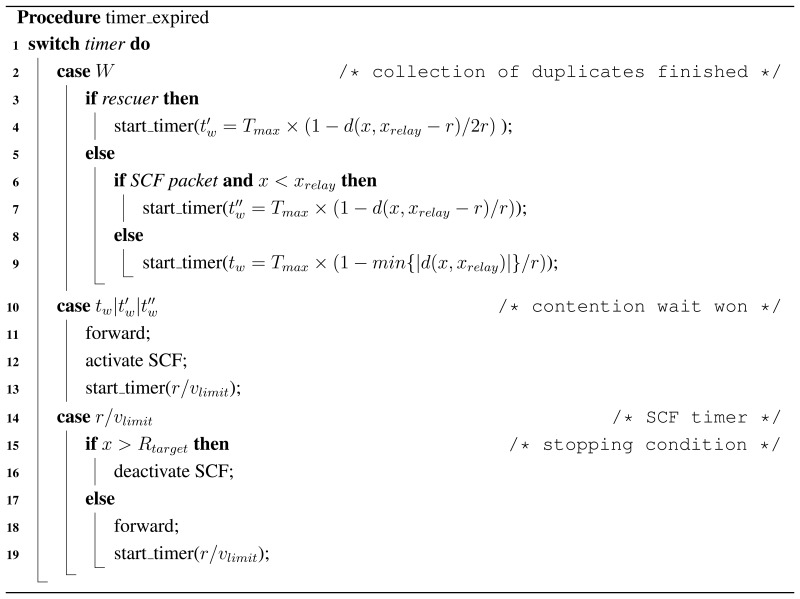


**Figure 9.**
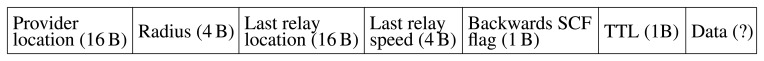
Message format.

The resulting message format is shown in Figure 9.

## 7. Evaluation

In this section, we are going to test by simulations the performance of the proposed solution in a variety of scenarios. First, we aim to validate the assumptions and equations from Section 4 in an ideal setting. Next, we will discover how much of an improvement we achieve by adding our store-carry-forward mechanism. Then, we will put the results in context by comparing them with a state-of-the-art proposal. We finish the evaluation by testing our solution in a more realistic scenario.

However, first of all, we offer a complete description of the simulation settings in the next subsection.

### 7.1. Scenario Settings

The vehicles in our simulations communicate via 802.11p technology, thanks to the extensions to ns-2 presented in [13]. We also make use of the Nakagami radio propagation model, suggested in [14], as the most suitable for VANETs. The configuration values for the radio propagation like in a highway (Highway 101 in the San Francisco Bay Area of the USA), as well as for 802.11p have been taken from the documentation released by the same authors.The resulting network characterization is contained in Table 1.

#### 7.1.1. Preliminary Tests

Before running the simulations, we needed to obtain values for *τ* and *W*. Parting from the resulting *τ*, we were able to define the optimal *T_max_*, as explained above.

The procedure to find *τ* was as follows. We put two fixed nodes together and another one 200 m apart, so that both of them were closer than *r* to it. The node that is alone sends a message, and the other two try to forward it according to the described scheme. As they are very close, both nodes forward the packet, because none of them can hear the other one's retransmission before trying to access the channel. We slowly moved only one of the receivers towards the sender in successive simulations. There is a point in which this node was able to abort its retransmission. The difference between the scheduled forwarding times at this point is *τ*. We were able to determine it with an error of one microsecond.

Finally, we have set up a scenario of 4 km with a traffic density of 30 vehicles/km. Every vehicle broadcasts unrelevant packets of 620 bytes to keep the channel loaded to 60%. In this scenario, one of them sends a message that is forwarded by the rest according to the described scheme. In such a congested network, packets collide with a high probability. We have observed the instants when any vehicle receives simultaneous duplicates of the same packet. The difference between the minimum and the maximum times is bounded. We set *W* to this bounding value.

The resulting set of values is listed in Table 2. We want to remark that they are true, as long as the simulations are configured as explained at the beginning of this section. If other configuration scripts are used, the proper values for *r*, *W*, *τ* and, consequently, *T_max_* could be different. If the same values were used in such circumstances, the performance of the solution would worsen. A bad value for *r* or *τ* could mean a longer delay or a higher number of simultaneous duplicates at each hop. Giving *W* a value that is too low could stop the dissemination at any point.

We have configured the simulations according to 802.11p and using a transmission power of 0.1 W. These values may vary in real life, due to a different configuration (of the antenna power, for example). If that were the case, it would be necessary to set the proper values of *r*, *τ* and *W*, which are dependent on the antennae and the MAC configurations.

#### 7.1.2. General Configuration

We model an interurban roadway according to [15]. We can assume an exponential distribution of the space between a vehicle and the next one at road level. This is more realistic in sparse traffic situations, but the authors point out that this is also a good approximation for dense traffic. Hence, the inter-vehicle space is exponentially distributed according to the traffic density, *ρ*, of each direction, so that the mean is 1/*λ* = 1/*ρ*.

We set a straight roadway in which vehicles move in both traffic directions. We consider the usual speed limits in the European Union—from 60 to 120km/h. Each vehicle is assigned its speed randomly, following a normal distribution.

In order to reduce the computational load, we have simplified the scenario to a half of it. The total length of the scenario is 6 km. The RSU is located at Km. 1 and the service target radius, *R_target_*, is set to 4km. The message data length for the simulations is 216 bytes. We chose this size based on the message format, explained later, in Section 6. The six first fields' length is fixed to 42 bytes, and the last one is variable. We expect that 174 characters are enough to specify the brand name and some prices. Together with the IP and UDP headers, the total packet size is 244 bytes. We want to remark that increasing the contents of the data field causes a little increase in the packet transmission time and the probability of collision. Every simulation batch is constituted by 100 runs, each using a different random scenario.

**Figure 10.**
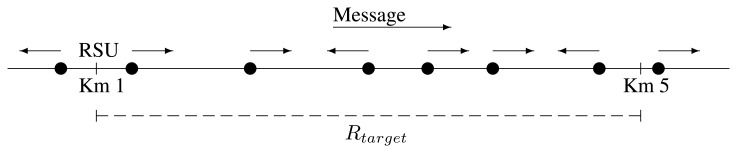
Simulations scenario.

These parameters are specified in Table 3. A visual representation of the simulations scenario is in Figure 10.

### 7.2. Testing the Distance-Based Forwarding Scheme

We test the base forwarding scheme in a fully connected scenario. We want to check if our assumptions and models are correct in an ideal setting (*i.e.*, with no other ongoing traffic).

#### 7.2.1. Scenario Settings

According to [15], again, we can assume that for a fully connected network, the global traffic density in a roadway segment is at least of 30 vehicles/km. Therefore, we use a range from 30 to 40 vehicles/km for these simulations. The traffic densities for each direction are symmetric.

Given that the traffic densities are high and vehicles will not be able to travel close to the upper limit, we set the mean speed at 90 km/h, with a standard deviation of 10 km/h.

We condense these data in Table 4.

#### 7.2.2. Results

**Figure 11.**
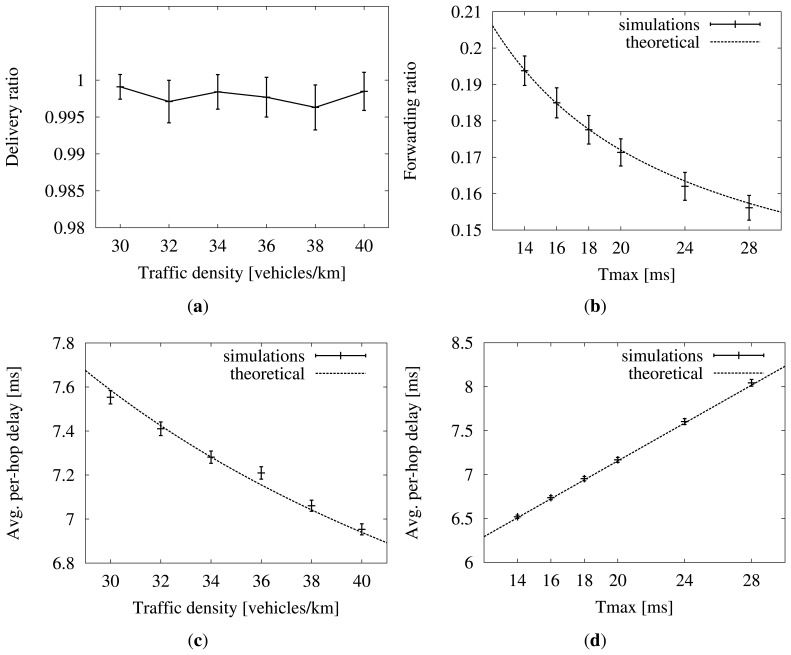
Simulation results, compared to theoretical values. Confidence interval of 95%. (**a**) Coverage (*T_max_* = 18 ms); (**b**) forwarders/receiver (*ρ* = 40 vehicles/km); (**c**) per-hop delay (*T_max_* = 18 ms); (**d**) per-hop delay (*ρ* = 40 vehicles/km).

After running the simulations, the first step is to check if they have reached a 100% coverage. A look at Figure 11a confirms that most of the runs conform to this requisite. Recall that the equations in Section 4 are only valid in connected networks.

In Figure 11b–d, we have drawn dotted lines to represent the theoretical forwarding ratio and average per-hop delay. The forwarding ratio is calculated with Equation (4). The average per-hop delay is easily obtained from Equation (1) by replacing the minimum distance with *E*[*d_max_*] and adding the fixed previous wait, *W*. We confirm that the values resulting from the simulations, drawn with error bars, tally with the analytical model. This proves that the suggested default value of *T_max_* = 18 ms, deduced from the mentioned equations, is appropriate for a moving scenario, too. The reason for this is that messages travel much faster than vehicles. Even if the relay had had to wait the maximum per-hop time (*i.e.*, 18 + 5 = 23 ms), the vehicle would have moved less than a meter from the reception of the packet to the time of forwarding it. Though in movement, the scenario is almost static from the point of view of communications speeds.

### 7.3. Testing the Store-Carry-Forward Mechanism

We have just proven the suitability of the base forwarding scheme. Now, we are going to test the solution, complete with our store-carry-forward proposal, against the forwarding scheme alone in sparse scenarios.

#### 7.3.1. Scenario Settings

The simulations are configured as explained in Section 7.1. We need to define some more parameters, summarized in Table 5.

In this case, we have set different traffic densities, from 5 vehicles/km (sparse) to 35 vehicles/km (dense) for one of the traffic directions, while maintaining the other fixed at 5 vehicles/km. This will help us visualize the influence of the population at each side of the road in the performance.

Again, the assigned speeds are related to the chosen traffic density for the scenario. That is, in a sparse traffic scenario, vehicles travel at speeds close to the upper limit (mean of 120 km/h), whereas in dense traffic scenarios, they are given lower speeds (mean of 90 km/h).

The complete set of scenario configurations for these tests is shown in Table 6.

#### 7.3.2. Results

**Figure 12.**
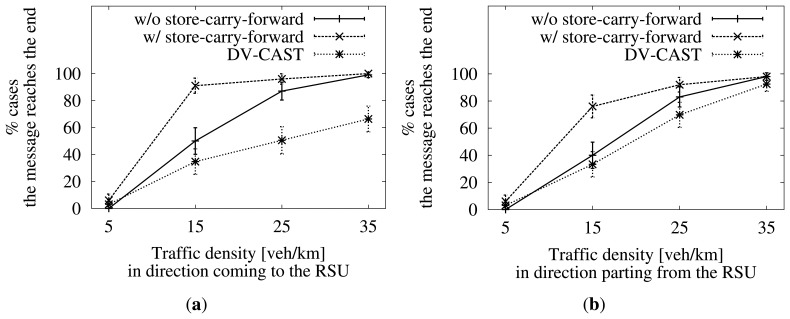
Success rate of our approach with and without store-carry-forward and of DV-CAST. Density variations in one direction influence the performance. The other direction has a fixed traffic density of five vehicles/km. Confidence interval of 95%. (**a**) Five vehicles/km in the direction parting from the roadside unit (RSU); (**b**) five vehicles/km in the direction coming to the RSU.

Let us recall that our RSU is located at kilometer 1 in the scenario, and it has set a target radius, *R_target_*, of 4 km. Therefore, the message should reach kilometer 5 thanks to the multi-hop dissemination carried out by the vehicles in the roadway. In Figure 12a,b, we can see the success rate in this task.

The first thing we can notice is that the addition of the store-carry-forward mechanism described here improves the results for cases of not full connectivity (less than 35 vehicles/km). Furthermore, despite its use, the results are still poor when the traffic density on both sides of the road is very low. Both aspects could be expected.

Now, we can see how the success rate, either when using store-carry-forward or not, is better when the traffic density in the direction approaching the RSU is higher. One would think that it should be better when the density in the opposite direction is higher. As these vehicles travel in the same direction as the message, it would have a better chance to get carried by them to the end of the target area. Therefore, this result is unexpected. We will come back to this later.

**Figure 13.**
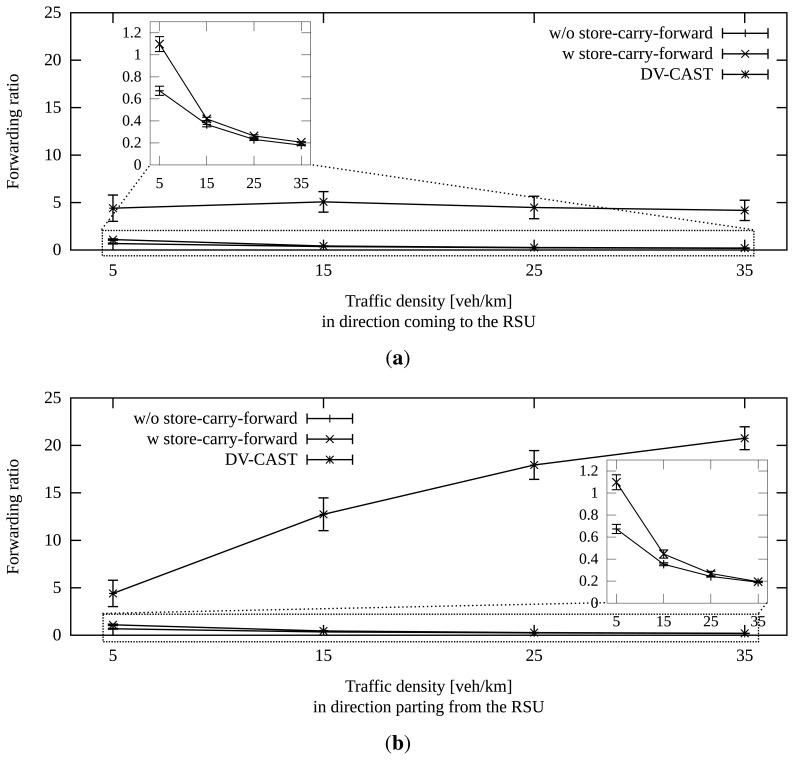
Overhead of our approach with and without store-carry-forward and of DV-CAST. Density variations in one direction influence the performance. The other direction has a fixed traffic density of five vehicles/km. Confidence interval of 95%. (**a**) Five vehicles/km in the direction parting from the RSU; (**b**) five vehicles/km in the direction coming to the RSU.

The general improvement in the success rate that provides store-carry-forward comes at the price of a higher number of messages. In Figure 13a,b, we can see the ratio of the number of duplicates per number of vehicles that heard the message. In the cases of extremely low density, this ratio is even greater than one. However, this is not a problem, because the total number of messages is still low.

There is a slightly lower ratio when the density of vehicles going away from the RSU is higher than 5%. This is due to one of the measures described above: though vehicles traveling towards the RSU are allowed to forward the message, the others are forced to become relays if they hear a vehicle from the former group store-carry-forwarding it. This increases the number of total duplicates in that dissemination. Furthermore, the higher number of retransmissions must be also the cause of the better success rate for this case.

### 7.4. Comparison with DV-CAST

We want to compare our solution with DV-CAST [10] as a state-of-the-art proposal. DV-CAST is a vehicular multi-hop broadcast for vehicular interurban environments, too. It implements its own store-carry-forward mechanism and can support distance-based flooding. The most obvious difference with our proposal is the use of periodic beacons to allow a general knowledge of the one-hop neighborhood.

#### 7.4.1. Scenario Settings

Along with the simulations in the previous section, we have run our implementation of DV-CAST in the same scenario. We have configured it as close to the description in [10] as possible. Only the propagation model (Rician fading) and the MAC protocol (802.11a) used in that work are different from ours (Nakagami and 802.11p, respectively). We have assigned reasonable values to parameters that were not explicitly claimed. We have used the same packet size as in our solution, though the headers are different, to make them as similar as possible. The complete set of values is shown in Table 7.

#### 7.4.2. Results

**Figure 14.**
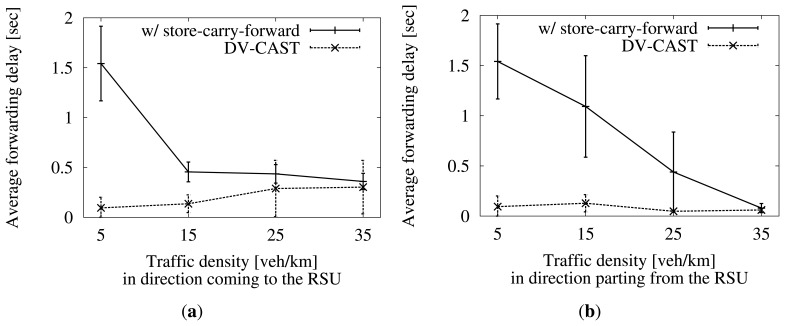
Average delay before forwarding at each hop by our proposal and by DV-CAST. Confidence interval of 95%. (**a**) Five vehicles/km in the direction parting from the RSU; (**b**) five vehicles/km in the direction coming to the RSU.

We can appreciate the differences visually in Figures 12, 13 and 14.

First, in Figure 12a,b, we show the success rate when trying to disseminate the message through the whole zone of interest (*R_target_*). DV-CAST achieves a lower rate, because its control over the existence of a new relay at each hop is minimum. In general, it relies on the information about one-hop neighbors and just expects that the message reached a vehicle that can forward it further. The authors of [11] agree in this conclusion.

Now, if we pay attention to Figure 13a,b, we can observe the number of packets sent by the number of receivers. This number is one when every receiver forwards every new packet (simple flooding). Let us recall that one of our main goals is to minimize it. However, the rate may increase when using store-carry-forward, because the same receiver may forward the same packet more than once. When using our proposal, we see this happen when the traffic density in both directions is very low. With DV-CAST, we see that a value greater than one is the general case. One reason for this is the slotted function to determine the next relay. More than one vehicle may concur in the same time slot. By using a continuous time function, our proposal reduces the probability of this situation.

We can also notice that this ratio is especially high for DV-CAST when the traffic density in the direction towards the RSU is low. The vehicles traveling in the opposite direction must always cooperate in the dissemination of the message. Therefore, they never tell if the message is new or already known to them. We have minimized the number of occasions when these vehicles take part in the dissemination of an old packet.

Finally, we see the average delay before forwarding at each hop in Figure 14a,b. Each vehicle has to decide whether to forward an incoming packet or not. In our proposal, this decision is based on the use of several timers, which add a delay. As the density gets higher, the probability of a vehicle ending the wait sooner gets higher, too. DV-CAST has a simpler approach, where a timer is needed only in the case of a well-connected scenario. Because of the reasoning above, the wait is short in DV-CAST, too. Thanks to this, the experienced delay is much lower than in our approach in sparse scenarios.

### 7.5. Performance under Background Traffic

We have run a final set of simulations to test the proposal in a more realistic scenario.

#### 7.5.1. Scenario Settings

The configuration is the same as in Section 7.3.1, but now, we add a channel load of 40% for a normal communications situation [16], as stated in Table 8. This channel load is assumed to be due to any type of communication that is already being carried out by all the vehicles in the scenario. We want to prove the performance of the proposed solution in a realistic scenario. Again, we have kept the traffic density in the direction coming to the RSU fixed at five vehicles/km, while varying the other direction's.

#### 7.5.2. Results

**Figure 15.**
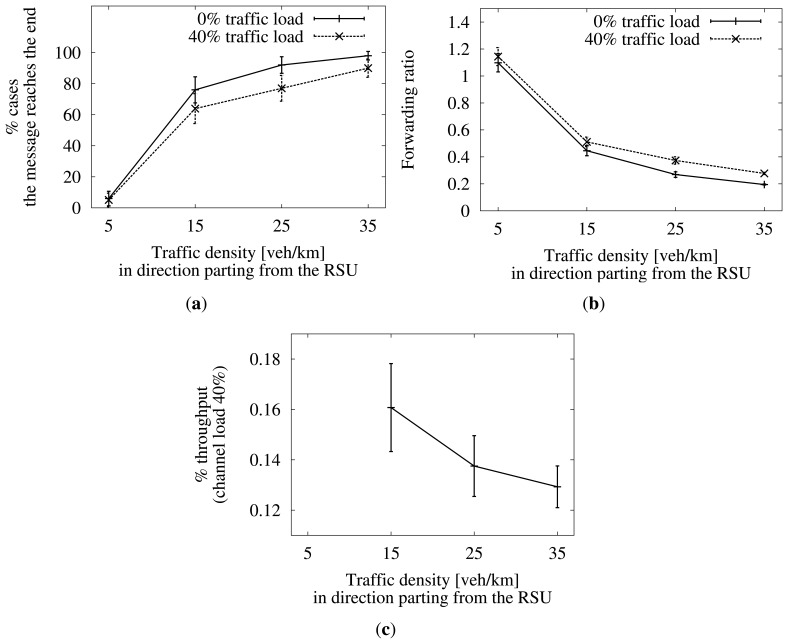
Performance when the wireless channel is loaded to 40%. Confidence interval of 95%.)

First, in Figure 15a, we see the comparison of the success rate when there is a channel load of 40% and when there is none. Also, in Figure 15b, we can see the number of generated duplicates by the number of vehicles that hear the message. Both parameters deteriorate when the channel is loaded, due to lost packets and collisions. Lost packets may prevent the message from reaching the end of the target area. If a relay hears the message from the next one too late, it may have already sent a new duplicate, increasing the forwarding ratio. If it misses the retransmission at all, it may even go on store-carry-forwarding the message until it leaves the target area.

On the other hand, we can see in Figure 15c that, despite the extra retransmissions, the percentage of the total throughput, due to the dissemination, is still low, around 0.15% at most. When the traffic density is very low, the dissemination stops soon, so this case is negligible and is not represented in the graphs. In addition, we can notice how the percentage gets smaller as the traffic density is higher. This is due to the lower number of retransmissions that comes from a higher probability of finding new neighbors at each hop.

## 8. Conclusions and Future Work

In this work, we have described a new service discovery solution for roadways. It will allow gas/charging stations to advertise their locations. This way, drivers can plan their next stop better on long trips, saving energy and CO_2_ emissions.

A simple approach, pushing short messages into a target radius, has proven to be enough for the gas station advertising system. In fact, the key of the system is a bandwidth-efficient flooding. Here, we have optimized our own previous proposal [1] and added a customized store-carry-forward mechanism in order to achieve this goal. We have tested the solution through extensive simulations and compared it to a state-of-the-art proposal, DV-CAST, with satisfactory results.

This solution is aimed at the planning of long trips and, hence, to a roadway environment. However, it could be useful for other purposes in a city scenario, too. As future work, we plan to do the necessary modifications in order to make it also suitable for a city environment.

Furthermore, we still have to select an algorithm that will sort the incoming announcements according to the user preferences.

## Figures and Tables

**Table 1.** Network parameters in the simulations.

**Parameter**	**Value**
Frequency band (10 MHz channel)	5.900 GHz
Propagation model	Nakagami
Transmission power	0.1 W
Antenna gain	1 dB
Sensitivity	−94 dBm
SNR	40 dBm
Thermal noise	−99 dBm
Communication range (*r*)	232 m
Encoding	OFDM
Modulation	BPSK
Bitrate	6 Mbps

**Table 2.** Simulation settings.

**Parameter**	**Value**
*r*	232 m
*τ*	0.914 ms
*W*	5 ms
*T_max_*	18 ms

**Table 3.** General simulation settings.

**Parameter**	**Value**
message length	216 bytes
scenario length	6 km
gas station position	Km. 1
*R_target_*	4 km
maximum speed limit (*v_limit_*)	120 km/h
simulation runs	100

**Table 4.** Simulation settings for testing the forwarding scheme.

**Parameter**	**Value**
speed distribution	∼N(90 km/h, 10 km/h)
traffic density (*ρ*)	30–40 vehicles/km

**Table 5.** Simulation settings.

**Parameter**	**Value**
maximum contention and propagation time	28 ms
*Δt_max_*	79 ms

**Table 6.** Scenario settings for testing the store-carry-forward mechanism.

**Traffic density (*ρ*)**	**Mean speed**	**SD**
5 vehicles/km	120 km/h	3.33 km/h
15 vehicles/km	120 km/h	3.33 km/h
25 vehicles/km	100 km/h	6.67 km/h
35 vehicles/km	90km/h	10km/h

**Table 7.** DV-CAST configuration.

**Parameter**	**Value**
*Hello* frequency	1 Hz
*Hello* packet size	61 bytes
Maximum neighbor table size	5
IP packet size	244 bytes
Packet Timer	2 min
Broadcast suppression	Slotted 1-persistence
*N_S_*	3 slots
*τ_DV–CAST_*	2 ms

**Table 8.** Simulation settings for testing the general performance.

**Parameter**	**Value**
channel load for ideal conditions	0%
channel load for normal conditions	40%

## A. Appendix

### A.1. Glossary

*Δ t_max_* sum of *W*, *T_max_* and the maximum time it takes the network to process the packet (collision resolution and propagation).

*d_guard_* guard distance to prevent vehicles close to the edge of the coverage range to take part in the forwarding process.

*d_min_* distance to the closest device from which this vehicle heard a duplicate of the same packet almost simultaneously.

*r* coverage radius of a vehicle's or RSU's antenna.

*ρ* traffic density in veh./km.

**RSU** fixed infrastructure with communication capabilities in a vehicular networking scenario.

*R_target_* radius of the area of interest specified in the message.

*τ* minimum difference between the delays calculated by two consecutive nodes, so that the second hears the first's transmission before attempting to forward.

*T_max_* maximum value for the forwarding delay *t_w_*.

*t_w_* delay a vehicle has to wait before forwarding a message in order to prioritize the vehicle most apart from the last relay.

*v_limit_* maximum legal speed in the roadway.

*W* short time during which a vehicle waits for almost simultaneous duplicates before computing a forwarding delay *t_w_*.

*x_relay_* denotes the current position of a relay.
